# OR7-005 – Canakinumab in childhood colchicine resistant FMF

**DOI:** 10.1186/1546-0096-11-S1-A106

**Published:** 2013-11-08

**Authors:** PJ Hashkes, Y Butbul Aviel, S Lubin, L Tseng, E Ben Dayan, T Rachmilewitz, R Brik

**Affiliations:** 1 Shaare Zedek Medical Center, Jerusalem, Israel; 2 Meyer Children's Hospital, Rambam Medical Center, Haifa, Israel; 3 Novartis Pharma Services AG, Petach Tikva, Israel; 4 Novartis Pharmaceuticals, East Hanover, NJ, USA

## Introduction

Familial Mediterranean Fever (FMF) is the most common hereditary autoinflammatory syndrome affecting >10,000 people in Israel. FMF is caused by mutations in the MEFV gene, which encodes for the pyrin protein that is part of the inflammation complex that activates IL-1β. Evidence from case reports/series and one controlled study supports IL-1 blockage as a potential treatment for FMF. Canakinumab (CAN) is a selective fully human monoclonal anti-IL-1β antibody.

## Objectives

This study served as a proof of concept to evaluate the role of CAN in the treatment of pediatric colchicine resistant (CR)-FMF.

## Methods

This was a 2-center open-label, single-arm study. The population consisted of CR FMF patients (pts) 4-20 years of age, with a history of at least 3 documented FMF attacks in the 3 months prior to enrollment.

Pts entered a 30-day run-in period (RI) during which FMF attacks were documented in a diary. Pts who experienced an investigator-confirmed FMF attack during RI were eligible to enter the treatment phase and receive a SC injection of CAN 2 mg/kg (max 150 mg) every 4 weeks for three times with the 1^st^ dose given during an attack. The dose was doubled to 4 mg/kg (max 300 mg) if an attack occurred between Day 1 and Day 29 visits. Following the end of the treatment period, pts were followed until Day 144 or until an attack occurred, whichever occurred first. Primary outcome was the proportion of pts with ≥50% reduction in FMF attack rate during the treatment vs. pretreatment period.

## Results

Fifteen Israeli pts (9 males, 6 females) with CR FMF entered the RI period and 7 (median age 9.5 yrs.; 6.8-14.9 yrs.), advanced to the treatment phase. In total, 6/7 (86%) pts had a ≥50% reduction in their FMF attack rate during the treatment period vs. the pretreatment period. The median attack rate was reduced by 89% from 2.7 per 28 days prior to CAN to 0.3 per 28 days during treatment; 2 pts had their CAN dose up titrated. Elevated median baseline CRP and SAA normalized by Day 8, ESR by Day 28 and all remained normal for remainder of trial. There was no evidence of neutralizing antibody formation. In all, 11 adverse events (AEs) in 4 pts were reported after the first CAN dose; all were mild except for 2 moderate AEs (Strep infection, laceration) assessed as unrelated to study treatment by the study investigator. No AEs led to medication discontinuation.

## Conclusion

In this study of pediatric pts with colchicine resistant FMF, canakinumab every 4 weeks substantially reduced the FMF attack rate, consistent with similar findings in adults. AEs were manageable. A larger study is needed to better evaluate the benefit of canakinumab in FMF.

## Disclosure of interest

P. Hashkes Grant / Research Support from: Novartis, Consultant for: Novartis, Speaker Bureau of: Novartis, Y. Butbul Aviel: None declared, S. Lubin Employee of: Novartis, L. Tseng Employee of: Novartis, E. Ben Dayan Employee of: Novartis, T. Rachmilewitz Employee of: Novartis, R. Brik Grant / Research Support from: Novartis

